# Dendritic Mesoporous Silica Hollow Spheres for Nano-Bioreactor Application

**DOI:** 10.3390/nano12111940

**Published:** 2022-06-06

**Authors:** Qian Zhang, Minying Wu, Yuanyuan Fang, Chao Deng, Hsin-Hui Shen, Yi Tang, Yajun Wang

**Affiliations:** 1 Department of Chemistry, Fudan University, Shanghai 200433, China; 13110220006@fudan.edu.cn (Q.Z.); 18110220035@fudan.edu.cn (M.W.); 19110220078@fudan.edu.cn (Y.F.); 2 College of Chemistry & Materials Engineering, Wenzhou University, Wenzhou 325027, China; dengchao@wzu.edu.cn; 3 Department of Materials Science and Engineering, Monash University, Clayton, VIC 3800, Australia; hsin-hui.shen@monash.edu

**Keywords:** hollow mesoporous silica nanoparticles, dendritic mesopores, co-deposition, barium sulfate, enzyme immobilization, bioreactors

## Abstract

Mesoporous silica materials have attracted great research interest for various applications ranging from (bio)catalysis and sensing to drug delivery. It remains challenging to prepare hollow mesoporous silica nanoparticles (HMSN) with large center-radial mesopores that could provide a more efficient transport channel through the cell for guest molecules. Here, we propose a novel strategy for the preparation of HMSN with large dendritic mesopores to achieve higher enzyme loading capacity and more efficient bioreactors. The materials were prepared by combining barium sulfate nanoparticles (BaSO_4_ NP) as a hard template and the in situ-formed 3-aminophenol/formaldehyde resin as a porogen for directing the dendritic mesopores’ formation. HMSNs with different particle sizes, shell thicknesses, and pore structures have been prepared by choosing BaSO_4_ NP of various sizes and adjusting the amount of tetraethyl orthosilicate added in synthesis. The obtained HMSN-1.1 possesses a high pore volume (1.07 cm^3^ g^−1^), a large average pore size (10.9 nm), and dendritic mesopores that penetrated through the shell. The advantages of HMSNs are also demonstrated for enzyme (catalase) immobilization and subsequent use of catalase-loaded HMSNs as bioreactors for catalyzing the H_2_O_2_ degradation reaction. The hollow and dendritic mesoporous shell features of HMSNs provide abundant tunnels for molecular transport and more accessible surfaces for molecular adsorption, showing great promise in developing efficient nanoreactors and drug delivery vehicles.

## 1. Introduction

Mesoporous silica nanoparticles (MSN) have attracted extensive research interest due to their high specific surface area, large pore volume, tunable particle morphologies and pore structures, and good biocompatibility [1,2]. MSNs of various shapes and morphologies, such as nanospheres [3,4], nanorods [5,6,7], nanocubes [8], nanotubes [9,10], and supraparticles [11], have been reported to meet the requirements for a range of applications, including drug delivery [12,13,14,15], bio-imaging [16], sensing [17,18], and catalysis [19,20,21]. Among them, hollow-structured MSNs (HMSN) are particularly attractive for nanoreactors and drug delivery applications, since their distinctive hollow cavities could serve as a reservoir to host a large number of guest molecules [22,23,24,25].

The most common way to prepare HMSN is to use a dual-template strategy, which involves introducing a sacrificial agent for the hollow structure and using a soft-template (usually a surfactant) to direct the formation of mesopores in the outer shell. Based on the nature of the hollow directing agent, the synthesis can be further divided into soft templating and hard templating approaches [26,27]. The soft templating method usually requires a large amount of surfactant, and it is difficult to control particle size and shell thickness. In contrast, the hard templating method has obvious advantages in overcoming these problems by applying prefabricated hard templates with precise control. Based on the physicochemical stability of silica, hard template agents used in HMSN preparation can usually be removed by acid etching, organic solvent dissolution, and calcination. Besides the hollow cavity, the texture structure of the pores in the shell is also an important parameter of the HMSN, as these mesopores can provide rich pathways to connect the exterior and interior environments. However, the HMSNs reported so far only possess rather tortuous and narrow mesoporous channels which are inefficient in the transport of guest molecules through the shell, since mass transport through the mesoporous shell is greatly influenced by the permeability of the shell [28,29,30].

Recently, Fu et al. reported a confined self-assembly method for the preparation of fractal silica nanoparticles with tetraethyl orthosilicate (TEOS), 3-aminophenol/formaldehyde, and ethylenediamine (EDA) in a surfactant-free sol-gel system [31]. The prepared fractal silica nanoparticles have a dendritic structure with large center-radial vertical mesopores. The co-deposition of 3-aminophenol-formaldehyde resin (APF) and silica is the key to forming such types of fractal silica nanoparticles. During the reaction, part of the TEOS added was to form silica core particles, which may limit the specific surface area of the final materials. This problem can be solved by designing an HMSN with only a dendritic silica shell [32].

**Figure 1:**
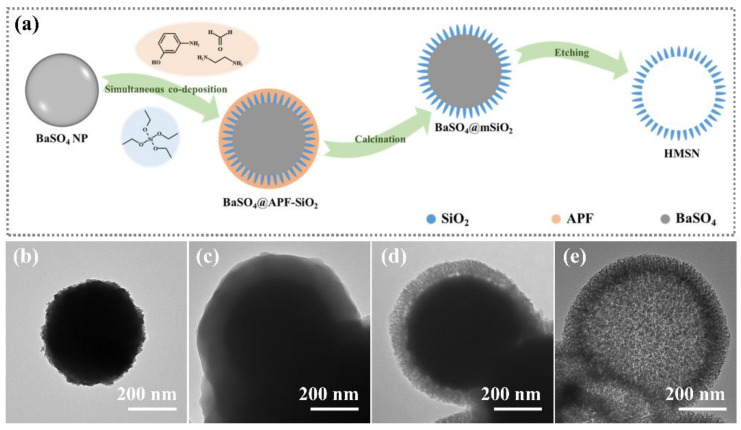
Schematic illustration of the strategy for the preparation of hollow mesoporous silica nanoparticles (HMSN) (**a**); transmission electron microscopy (TEM) images (**b**–**e**) of BaSO_4_ NP, BaSO_4_@APF-SiO_2_, BaSO_4_@mSiO_2_, and HMSN, respectively.

In this work, we propose a novel method to prepare HMSNs with a dendritic mesoporous shell. The synthesis is achieved through the sol-gel co-deposition of silica and APF onto the surface of barium sulfate nanoparticles (BaSO_4_ NP), which act as hard templates for the hollow cavity (Figure 1a). The advantages of using BaSO_4_ NP as the sacrificial core template are that it is easy to prepare, size-adjustable, stable in water, and easy to remove with weakly alkaline solutions of ethylenediaminetetraacetic acid (EDTA) [33]. Also, the surface of BaSO_4_ NPs is full of OH^−^, which provides an affinity interface for the sol-gel coating of silica [34]. The produced HMSN has a large hollow cavity, a dendritic silica shell, and broad, open, and penetrating mesoporous channels. Furthermore, the pore structure and morphology of the HMSN can be tuned by adjusting the amount of TEOS added and using BaSO_4_ NPs of different sizes as templates. As a proof-of-principle study, the immobilization of catalase in HMSNs has been carried out to demonstrate its application potential as a nanoreactor.

## 2. Materials and Methods

### 2.1. Materials

Ethanediamine (EDA, 99%), formaldehyde (30–40%), ethyl alcohol (99.7%), 3-aminophenol (99%), ethylenediamine tetraacetic acid disodium salt (EDTA·2Na, 99%), H_2_SO_4_ (95–98%), HCl (36–38%), NH_3_·H_2_O (25–28%), and BaCl_2_·2H_2_O (99.5%) were purchased from Sinopharm Chemical Reagent (Shanghai, China). NaOH (96%) and Na_2_SO_4_ (99%) were purchased from Shanghai Dahe Chemicals (Shanghai, China). NaH_2_PO_4_·2H_2_O (99%), and Na_2_HPO_4_·12H_2_O (99%) were purchased from Chinasun Specialty Product (Changshu, China). Tetraethoxysilane (TEOS, 99%), catalase (from bovine liver, 2000–5000 units/mg protein), 3,3′,5,5′-tetramethylbenzidine (TMB, 98%), and peroxidase from horseradish (HRP, ~200 units/mg) were purchased from Sigma–Aldrich (St. Louis, MO, USA). All chemicals were used without further purification.

### 2.2. Synthesis of Barium Sulfate Nanoparticles

First, a Ba-EDTA solution of 0.125 mol L^−1^ was prepared by mixing BaCl_2_ solution and EDTA·2Na solution with equal moles. Under vigorous stirring, 0.5 mL of Na_2_SO_4_ aqueous solution (0.5 mol L^−1^) was added rapidly to 2.0 mL of Ba-EDTA solution at pH 6.0. After standing for 1 h, the precipitation was separated by centrifugation at 8500 rpm for one minute, washed three times with deionized water, and then dried at 70 °C. The sample obtained was denoted as BaSO_4_ NP.

### 2.3. Synthesis of Hollow Mesoporous Silica Nanoparticles

In a typical synthesis, 1.0 g of BaSO_4_ NP was dispersed in 100 mL of ethanol solution. Then, 25 mL of deionized water, 3.9 mL of concentrated ammonia, 560 μL of ethylenediamine, and 1.025 g of 3-aminophenol were added. After that, 2.25 mL of formaldehyde solution and different amounts (0.545, 1.095, and 2.190 mL) of TEOS were added under magnetic stirring. The reaction was carried out at room temperature for 4 h. After centrifuging separation and washing with a 1:1 ethanol–water mixed solution, the sample was dried in an oven at 70 °C to obtain a pale yellow powder (BaSO_4_@APF-SiO_2_). After calcinating the BaSO_4_@APF-SiO_2_ in an air atmosphere to remove the organic species, mesoporous silica-coated BaSO_4_ NP (BaSO_4_@mSiO_2_) was obtained. The heating rate was 5 °C min^−1^, and the temperature was maintained at 600 °C for 6 h. Finally, the hollow mesoporous silica nanoparticles (HMSNs) were obtained after the removal of the BaSO_4_ core in a pH 9.0 EDTA solution. The final products with different amounts of TEOS addition (0.545, 1.095, and 2.190 mL) in the synthesis were denoted as HMSN-0.55, HMSN-1.1, and HMSN-2.2, respectively. For comparison, solid mesoporous silica nanoparticles (SMSN) were also prepared with a similar experimental method to HMSN-1.1 except that the hard templating agent, barium sulfate nanoparticle, was not added to the system.

### 2.4. Catalase Immobilization and Activity Assay

A sodium phosphate buffer solution (50 mmol L^−1^) at pH 7.0 was used for enzyme immobilization experiments. The concentration of each kind of silica nanoparticle was 0.5 mg mL^−1^, while the initial concentration of catalase was 0.125, 0.25, and 0.50 mg mL^−1^, and the adsorption was carried out on a thermostatic oscillator for 4 h at 20 °C. After centrifugation (8000 rpm, 5 min), the absorbance of the supernatant was measured at 278 nm by a UV-vis spectrophotometer, and the catalase concentration in the supernatant was calculated using the standard curve as shown in Figure S1. The enzyme immobilization amount was calculated accordingly.

The enzymatic activity of catalase was monitored by the catalytic decomposition of H_2_O_2_. First, H_2_O_2_ (0.2 mmol L^−1^) was added to a phosphate-buffered solution (PBS, 50 mmol L^−1^, pH 7.0, 40 °C) with a catalase concentration of ~7.5 μg mL^−1^. Then, 50 μL of reaction solution was withdrawn every other minute and added to 2 mL of PBS containing 10 μg of HRP and 0.1 mg of TMB, which was incubated for 30 min at 40 °C and terminated by 50 μL of 2 mol L^−1^ H_2_SO_4_. The absorbance was measured at 450 nm to track changes in H_2_O_2_ concentration [35,36]. The standard curve was shown in Figure S2.

### 2.5. Characterization

Scanning electron microscopy (SEM) images were obtained by high-resolution transmission electron microscopy (Zeiss Ultra 55, Carl Zeiss, Jena, Germany) at 5 kV. Transmission electron microscopy (TEM) images were collected by a Hitachi HT7800 transmission electron microscope (Tokyo, Japan) operating at 100 kV and an FEI Tecnai G2 F20 S–Twin transmission electron microscope (Hillsboro, OR, USA) at 200 kV. The high-angle annular dark field scanning transmission electron microscopy (HAADF-STEM) image and energy dispersive X-ray spectroscopy (EDX) element mappings were achieved by the FEI instrument. X-ray diffraction (XRD) patterns were obtained using Cu-K radiation (λ = 1.54056 Å) with a tube voltage of 5 kV and a current of 30 mA on a Bruker AXS D2 PHASER diffractometer (Karlsruhe, Germany). Fourier transform infrared (FTIR) spectra measurements were conducted on a Spectrum Two spectrometer (Perkin–Elmer, Waltham, MA, USA) with an attenuated total reflectance cell. N_2_ sorption experiments were carried out on a Quadrasorb apparatus from Quantachrome Instruments (Boynton Beach, FL, USA). The samples underwent a degas process of 300 ℃ for 3 h. The multi-point Brunauer–Emmett–Teller (BET) method was used to calculate the specific surface areas with 9 points in the p/p_0_ range of 0.04–0.28. The mesopore volumes were determined at p/p_0_ = 0.989 and pore size distributions were derived from the desorption branch of the N_2_ sorption isotherm using the Barrett–Joyner–Halenda (BJH) model. A thermogravimetry (TG) analysis was performed on a synchronous thermal analyzer (SDT Q600, New Castle, DE, USA) heated from 30 °C to 900 °C at a rate of 10 °C min^−1^ under an air atmosphere. The UV-vis spectra were obtained with a Shimadzu UV-2600 spectrophotometer (Kyoto, Japan).

## 3. Results and Discussion

Figure 1a gives an illustration of the strategy proposed for the preparation of HMSN. In this strategy, spherical BaSO_4_ NP is used as the sacrificial template for the inner cavity, and the APF serves as the mesoporous template and structural direct agent in the shell. The monodispersed BaSO_4_ NP is prepared through a controlled deposition process based on the literature report [33]. The key to this synthesis is that EDTA was added to chelate with Ba^2+^ and control the release of Ba^2+^ in solution. As a result, Ba^2+^ and SO_4_^2−^ precipitate in a more controlled manner, allowing for the optimization and preparation of monodisperse and homogeneous spherical particles (Figure 1b and Figure S3b).

The formation of the HMSN was studied through tracking morphology changes of the intermediate particles by TEM (Figure 1b–e). BaSO_4_ NP with an average particle size of 368 nm (RSD 22%) was first prepared as the sacrificial template here. The particles were dispersed in an ethanol/water mixture and then adjusted with an ammonium hydroxide solution. Following the addition of formaldehyde and 3-aminophenol, EDA, and TEOS, the solution changed from white to pale yellow slowly as the reaction progressed. During this process, a synergistic formation of APF oligomers (through polycondensation from 3-aminophenol and formaldehyde) and silica precursors (through the hydrolysis of TEOS) in a basic solution was achieved. The formed APF and silica were co-deposited on the surface of the BaSO_4_ NP gradually, thereafter forming a hybrid APF–SiO_2_ shell coated on the BaSO_4_ NP (Figure 1c). After removal of the organic species by calcination, mesoporous silica-coated BaSO_4_ NP (BaSO_4_@mSiO_2_) was obtained. As shown in Figure 1d, the BaSO_4_@mSiO_2_ has a BaSO_4_ NP core with a mesoporous silica shell. After the dissolution of BaSO_4_ using a weakly alkaline EDTA solution, HMSN was finally obtained. As shown in Figure 1e, the HMSN possesses a tree-branch-like dendritic silica shell and a large interior cavity.

**Figure 2:**
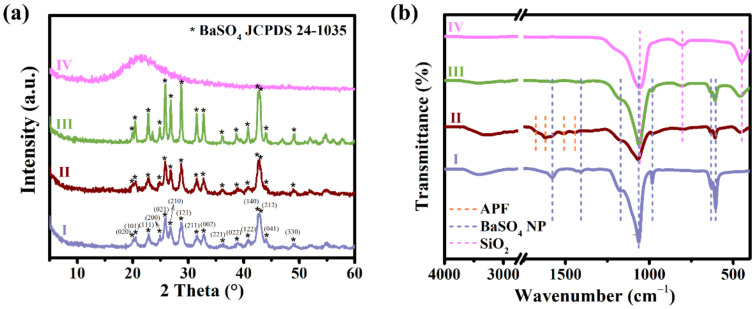
X-ray diffraction (XRD) patterns (**a**) and Fourier transform infrared spectroscopy (FTIR) spectra (**b**) of (I) BaSO_4_ NP, (II) BaSO_4_@APF-SiO_2_, (III) BaSO_4_@mSiO_2_, and (IV) HMSN, respectively.

XRD measurements have also been used to verify the HMSN preparation, as shown in Figure 2a. Peaks that correspond to an orthogonal crystal BaSO_4_ (JCDPS No. 24–1035) exist in the patterns of BaSO_4_ NP, BaSO_4_@APF-SiO_2_, and BaSO_4_@mSiO_2_. These peaks totally disappear in the XRD pattern of HMSN, indicating the successful removal of BaSO_4_ with the EDTA solution. The broad peak that occurs in the pattern of HMSN could be attributed to amorphous silica.

In order to monitor component changes of the samples in the synthetic process, the FTIR spectra of BaSO_4_ NP, BaSO_4_@APF-SiO_2_, BaSO_4_@mSiO_2_, and HMSN, were detected. The characteristic peaks of BaSO_4_ occurred at 1171, 1063, 982, 632, and 605 cm^−1^ (Figure 2b) [33,34]. Two additional peaks at 1577 and 1407 cm^−1^ could be assigned to the asymmetric and symmetric stretching vibrations of C–O in ionized and coordinated EDTA, suggesting that some EDTA coexists with the BaSO_4_ NP [37,38]. All the above peaks could be figured out in the spectra of BaSO_4_ NP, BaSO_4_@APF-SiO_2_, and BaSO_4_@mSiO_2_, but disappeared in the spectrum of HMSN, which confirms the complete removal of BaSO_4_. In the spectrum of BaSO_4_@APF-SiO_2_, the peaks of APF could be recognized, which are at 1676 (C=N/C=C stretching vibration), 1619 (benzene skeleton stretching vibration), 1508 (benzene skeleton stretching vibration), 1442 (benzene skeleton stretching vibration/C–H bending vibration), and 1349 (C–N stretching vibration) cm^−1^ [39]. The peaks at 1056, 863, and 449 cm^−1^, which are assigned to asymmetric stretching, symmetric stretching, and bending vibration of Si–O–Si bonds, respectively, appear in the spectra of BaSO_4_@APF-SiO_2_, BaSO_4_@mSiO_2_, and HMSN [40].

**Figure 3:**
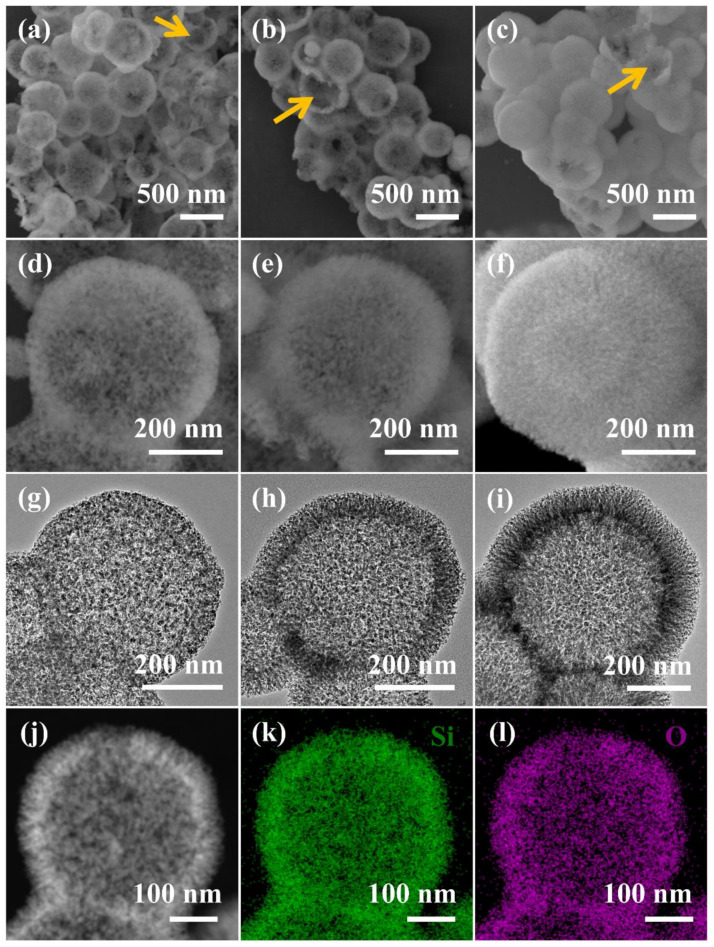
Scanning electron microscopy (SEM) images (**a**–**f**) at different magnifications and TEM images (**g**–**i**) of HMSN-0.55 (**a**,**d**,**g**), HMSN-1.1 (**b**,**e**,**h**), and HMSN-2.2(**c**,**f**,**i**), respectively. High angle angular dark field-scanning transmission electron microscopy (HAADF-STEM) image (**j**) and energy dispersive X-ray (EDX) element mappings (**k**,**l**) of HMSN-1.1. The orange arrows in (**a**–**c**) point to hollow cavity in the fragmentized HMSNs.

The morphology of the silica branches, as well as the pore structure, can be tuned by altering the TEOS addition amount (Figure 3). The final products are named HMSN-0.55, HMSN-1.1, and HMSN-2.2 according to the TEOS addition. The HMSN mentioned above is specifically referred to as HMSN-1.1. The hollow cavity of the particles can be confirmed by the fragmentized HMSNs, as indicated by the orange arrows in the SEM images in Figure 3a–c. The silica branches’ morphology can be distinguished from the SEM images in Figure 3d–f, where HMSN-2.2 is more densely branched than the others who possess wider slits in the branch crowd. The hollow dendritic structure can be clearly observed from the TEM images in Figure 3g–i, and HMSN-1.1 and HMSN-2.2 have more obvious tree-branch-like shells with through pores between the silica branches, while the HMSN-0.55 has relatively unordered and partially penetrated pores in the shell. Since the BaSO_4_ NP of the same size is used, the inner diameters of all three samples are close to each other. The shell thickness was measured to be 52.0 ± 3.4 nm, 67.5 ± 4.1 nm, and 92.0 ± 7.7 nm for HMSN-0.55, HMSN-1.1, and HMSN-2.2, respectively. The HAADF-STEM image and the EDX element mappings of HMSN-1.1 have been measured. The element components of Si and O, and their uniform distribution, are well confirmed by Figure 3j–l. The EDX spectroscopy of HMSN-1.1 is shown in Figure S4, in which only silicon and oxygen signals exist besides the background of the Cu mesh and carbon membrane signals, demonstrating the removal of APF and BaSO_4_ from HMSN.

**Figure 4:**
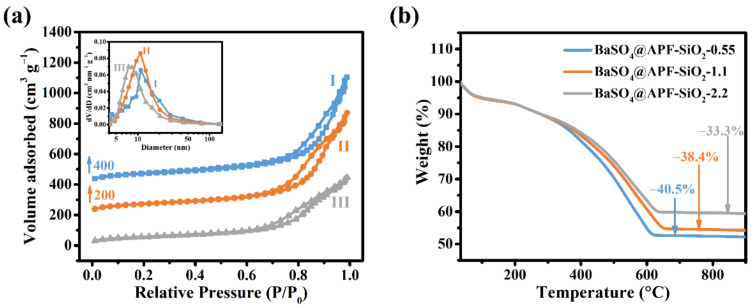
(**a**) N_2_ sorption isotherms of (I) HMSN-0.55, (II) HMSN-1.1, and (III) HMSN-2.2; the inset is the Barrett–Joyner–Halenda (BJH) pore distributions derived from desorption branches, (**b**) Thermogravimetry (TG) curves of BaSO_4_@APF-SiO_2_ prepared with TEOS additions of 0.545, 1.095, and 2.190 mL.

As shown in Figure 4a, nitrogen sorption isotherms have been measured to study the texture properties of HMSNs. All samples show type IV curves and a capillary condensation-induced hysteresis loop at relative pressures (P/P_0_) of around 0.75–0.98, 0.70–0.94, and 0.67–0.90 for HMSN-0.55, HMSN-1.1, and HMSN-2.2, respectively. The lowering of the pressure of the hysteresis loop signifies the decrease of the mesopore size. The texture properties of HMSN-0.55, HMSN-1.1, and HMSN-2.2 are summarized in Table 1. HMSN-0.55 has a similar BET specific surface area (255 m^2^ g^−1^) to HMSN-1.1 (257 m^2^ g^−1^) and is larger than HMSN-2.2 (200 m^2^ g^−1^). The BJH pore size distributions of HMSN-0.55, HMSN-1.1, and HMSN-2.2 are wide, centered at 11.0, 10.9, and 7.5 nm, respectively, and decrease as the amount of TEOS used in the synthesis increases (Figure 4a-inset). HMSN-0.55 and HMSN-1.1 have close pore volumes and average pore sizes that are both significantly larger than HMSN-2.2.

As the APF is performed as a template for the formation of mesopores in shell, the thermogravimetry (TG) analysis has also been used to analyze the proportions of APF in the BaSO_4_@APF-SiO_2_ hybrid particles. As shown in Figure 4b, the weight losses of BaSO_4_@APF-SiO_2_ prepared with TEOS additions of 0.545, 1.095, and 2.190 mL are 40.5, 38.4, and 33.3%, respectively, at temperatures ranging from 200 to 600 °C. The results suggest that the organic proportion (porogen) in BaSO_4_@APF-SiO_2_ is decreased as the amount of TEOS increases in the synthesis. These data are consistent with the decrease in pore volumes from 1.12 to 0.72 cm^3^ g^−1^ with increasing TEOS addition from 0.545 to 2.109 mL.

**Figure 5:**
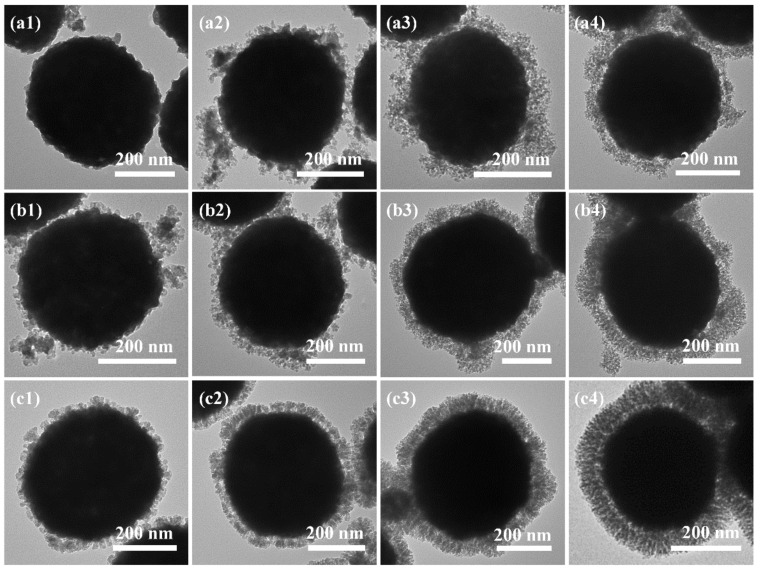
TEM images of the calcined BaSO_4_@APF-SiO_2_ prepared with different shell growth time: 15 min (**a1**,**b1**,**c1**), 30 min (**a2**,**b2**,**c2**), 60 min (**a3**,**b3**,**c3**), and 120 min (**a4**,**b4**,**c4**). The a–c series of samples correspond to a TEOS addition of 0.545, 1.095, and 2.190 mL, respectively.

The evolution of the BaSO_4_@APF-SiO_2_ was further studied through tracking the morphology changes of the intermediate particles by TEM (Figure S5). The calcined samples were also subsequently analyzed for a better observation of the mesoporous shell structure (Figure 5). The formation of the core–shell BaSO_4_@APF-SiO_2_ is an EDA-directed co-assembly process [41]. Through comparison of Figure S5 and Figure 5, it is clear that the silica species and APF species are deposited synchronously rather than sequentially in the reaction system.

A growth mechanism was proposed as follows. First, TEOS is hydrolyzed, heterogeneously nucleated, and grown into silica primary nanoparticles (SPNPs). Then, the SPNPs assemble with APF oligomers and are simultaneously deposited onto the surface of BaSO_4_ NP. The deposition process starts in a random manner with the formation of an incomplete shell, which then gradually grows into a uniform layer that becomes thicker and thicker as the reaction proceeds. Besides the longer reaction time, additional reactants can also improve the random and uneven deposition. Specifically, a limited TEOS addition (0.545 mL) is deposited more randomly and grows into an uneven layer. In the beginning, only a few SPNPs form an incomplete layer (Figure 5a1), especially in the very inner shell. It improves slightly as reaction time increases (Figure 5a2–a4). Moderate TEOS addition (1.095 mL) could form more SPNPs, which are deposited more uniformly as a denser layer (Figure 5b1–b4). However, if too much TEOS (2.190 mL) is added, the outer shell becomes too dense, resulting in thicker silica branches and lower pore volumes (Figure 5c1–c4).

Based on the above analysis, as the TEOS addition is increased, the generation rate, number, and size of SPNPs are increased, resulting in more dense silica branches and a larger proportion of silica in the outer shell of BaSO_4_@APF-SiO_2_. Thus, the proportion of porogen (APF template) is reduced in the shell, as confirmed by the TG curves in Figure 4b. As a result, the specific surface area, pore volume, and mesopore size of HMSNs are also reduced with the increase of TEOS added (Table 1). As shown in Figure 3h–i, HMSN-1.1 and HMSN-2.2 have apparent spiky shapes, reflecting their dendritic pore structures in the shell. However, the spiky shape is not evident in HMSN-0.55 (Figure 3g), probably because the addition of a small amount of TEOS (0.545 mL) formed fewer and smaller SPNPs that were insufficient to develop into the spike. In this case, the scattered deposition of the assembled SPNPs and APF oligomers causes a disordered network of mesopores with a wider pore distribution.

**Figure 6:**
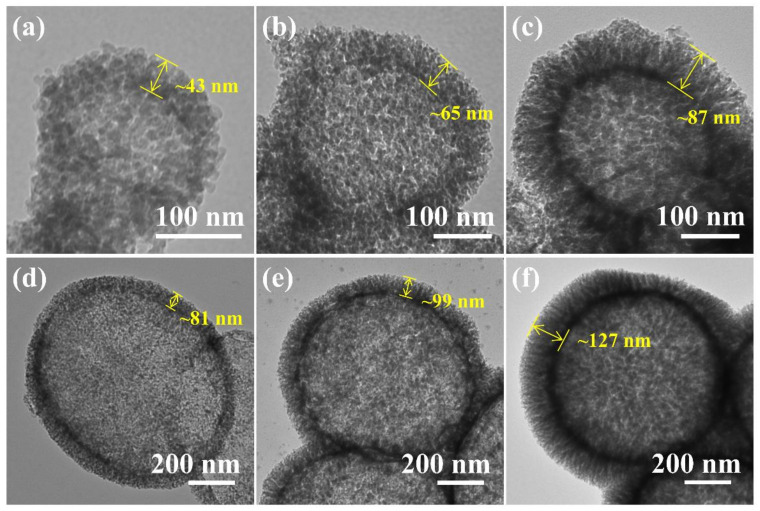
TEM images of HMSNs synthesized with BaSO_4_ NP-S (**a**–**c**) and BaSO_4_ NP-L (**d**–**f**) as the sacrificial template. The amount of TEOS added in the synthesis was 0.545 mL (**a**,**d**), 1.095 mL (**b**,**e**), and 2.190 mL (**c**,**f**), respectively.

In addition to the TEOS amount, size of the BaSO_4_ NP template also has a great effect which influences not only the diameter of the cavity but also the morphology of the silica branches. We synthesized barium sulfate nanoparticles in smaller (BaSO_4_ NP-S) and larger (BaSO_4_ NP-L) sizes by modifying the synthesis conditions (described in Supporting Information). As shown in Figure S3a,c, the average sizes of BaSO_4_ NP-S and BaSO_4_ NP-L are 183 nm (RSD 27%) and 749 nm (RSD 24%), respectively. A series of HMSNs were prepared with different TEOS additions and using BaSO_4_ NP-S and BaSO_4_ NP-L as the sacrificial templates. The materials all have a hollow structure and a mesoporous shell (Figure 6). As the particle size of BaSO_4_ NP increases, the cavity diameter of the HMSN also increases. An increase in the BaSO_4_ NP particle size can also increase the shell thickness of HMSNs. When the same weights of different BaSO_4_ NPs are used, the larger particles have a smaller specific surface area, resulting in a thicker shell being formed on them. In addition, the larger surface area of smaller particles has a similar effect as a small amount of TEOS added, which, as analyzed above, leads to more random assembly rather than dendritic silica shells. As a result, when BaSO_4_ NP-S is adopted, the spiky shape only appears at a TEOS addition of 2.190 mL (Figure 6c), but when BaSO_4_ NP-L is used, distinct branches can be observed regardless of whether the TEOS addition was 0.545, 1.095, or 2.190 mL (Figure 6d–f).

Enzyme immobilization has received a lot of attention since immobilization would provide an enzyme with improved stability, reusability, and control over reactions, as well as lower economic expenses [42]. As the HMSNs possess a hollow inner cavity and a unique dendritic mesoporous shell, application of the HMSNs as nanoreactors for enzyme-catalyzed reactions was investigated, and the immobilization of catalase was chosen as a proof-of-principle study. Catalase, with a molecular weight of 250 kDa and a size of 7 nm × 8 nm × 10 nm, is an antioxidant enzyme that catalyzes the conversion of dangerous hydrogen peroxide to nontoxic water and molecular oxygen used in the food industry [43].

For comparison, solid mesoporous silica nanoparticles (SMSN) have also been prepared by the same procedures as for HMSN-1.1 preparation, excepting there was no usage of BaSO_4_ NP as the core template. The TEM image (Figure S6a) reveals a dendritic, porous structure of SMSN. As shown in Figure S6b, SMSN has an N_2_ sorption isotherm with a type IV curve and an H2 hysteresis loop. As summarized in Table 1, it has a specific surface area of 212 m^2^ g^−1^, significantly smaller than HMSN-0.55 and HMSN-1.1 but slightly larger than HMSN-2.2. The pore volume of SMSN (0.52 cm^3^ g^−1^) is apparently smaller than all of the HMSNs, and it is only around half of the pore volume of HMSN-0.55 and HMSN-1.1. The pore size distribution of SMSN is narrower, centered at around 6.8 nm.

**Figure 7:**
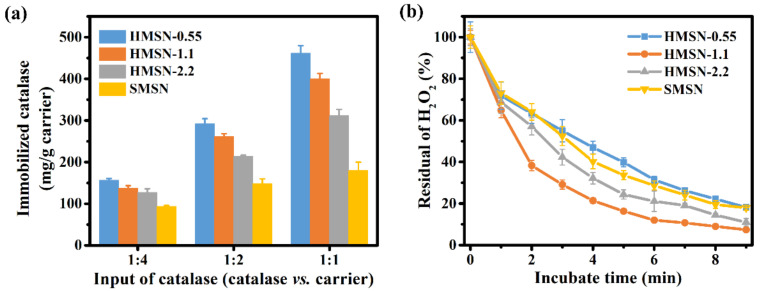
(**a**) Amount of catalase immobilized on various mesoporous silica materials with a catalase/carrier ratio of 1:4, 1:2, and 1:1 in the initial incubation solution. Concentration of the carriers was fixed at 0.5 mg g^−1^. (**b**) Comparison of catalase activity in H_2_O_2_ decomposition for the enzymes immobilized in HMSNs and SMSN.

As shown in Figure 7a, the immobilized amount of catalase increases on all carriers with the increase in the mass ratio of catalase/carrier. In general, the catalase loading capacity of the carrier decreases in the order of HMSN-0.55, HMSN-1.1, HMSN-2.2, and SMSN, and their difference in the loading capacity enlarges with the catalase/carrier mass ratio increase. When the equal weight of catalase is added, HMSN-0.55, HMSN-1.1, HMSN-2.2, and SMSN have a loading of 462, 400, 312, and 180 mg per gram of carrier, respectively. The higher total pore volume of HMSN-0.55 may account for its high immobilization capacity which allows it to contain more catalase in the confined pores. Though SMSN has a larger specific surface area than HMSN-2.2, the loading capacity is significantly lower than all three HMSNs. The lower loading capacity of SMSN may be attributed to the absence of the inner cavity and the much smaller pore size and pore volume of the mesoporous particles. These data suggest that the superior loading capacity of HMSNs could be ascribed to the dendritic mesoporous channels and the hollow inner cavity which provide a more accessible surface for enzyme immobilization.

The enzymatic activity of catalase is evaluated through the decomposition reaction of H_2_O_2_. The residual H_2_O_2_ has been monitored in reactions catalyzed by catalase immobilized on different carriers. For ease of comparison, the HMSN-0.55, HMSN-1.1, HMSN-2.2, and SMSN are loaded with similar catalase amounts of 147.5, 134.9, 125.5, and 139.0 mg g^−1^, respectively. As shown in Figure 7b, catalase immobilized in HMSN-1.1 showed the fastest catalytic decomposition rate. Despite having a similar specific surface area and porosity to HMSN-1.1, the catalase immobilized in HMSN-0.55 has a much lower enzymatic activity, likely caused by the randomly distributed pores and tortuous diffusion paths in its shell that limit the molecular transport. The SMSN possesses a dendritic pore structure, but the activity of catalase immobilized in it is lower than that of HMSN-1.1 and HMSN-2.2. The SMSN has much longer (about 176.0 nm) pore channels than HMSN-1.1 (about 67.5 nm) and HMSN-2.2 (about 92.0 nm), estimated from the thickness of the silica shell. The longer diffusion pathway, together with the smaller pore size and pore volume, are all responsible for the less-efficient performance of catalase immobilized in SMSN. The specific enzyme activities have also been measured, as shown in Figure S7b,c. The catalase loaded in HMSN-0.55, HMSN-1.1, HMSN-2.2, and SMSN, and free catalase have enzyme activities of 1016, 1331, 1027, 1121, and 2123 U mg^−1^, respectively. The enzyme activity of catalase was kept at 48–63% after loading. The enzyme was loaded though electrostatic interaction with minimum influence on the enzyme configuration. At the same time, blocking of the active sites after enzyme loading in a confined environment would reduce the enzyme activity [44,45,46]. Catalase loaded in HMSN-1.1 has the best-preserved enzyme activity (63%), suggesting that catalase loaded in HMSN-1.1 has more accessible active sites. The results suggested that the high specific surface area, large pore volume, distinct dendritic shell, and hollow structure of HMSN-1.1 are beneficial to the immobilized catalase for achieving high activity.

## 4. Conclusions

In summary, we have proposed a novel method to prepare HMSNs with a highly accessible mesoporous shell through combining the BaSO_4_ NP as the hard template and the in situ-formed APF as porogen for directing mesopores formation. HMSNs with different particle sizes, shell thicknesses, and pore structures have been prepared by choosing BaSO_4_ NPs with different sizes and adjusting the amount of TEOS added in synthesis. The HMSN-1.1 has a high specific surface area (257 m^2^ g^−1^), a large pore volume (1.07 cm^3^ g^−1^), a large pore size (10.9 nm), and dendritic mesopores penetrated through the silica shell. Owing to the unique hollow structure and a dendritic mesoporous shell, HMSNs showed significantly higher (over two folds) loading capacity for catalase immobilization than the counterpart SMSN prepared without the BaSO_4_ NP template. Advantages of HMSN with a dendritic mesoporous shell are also demonstrated from the catalase-loaded nanoreactors with superior enzymatic activity. The highly porous and dendritic mesoporous shell features of HMSNs hold enormous promise in developing more efficient nanoreactors and drug delivery vehicles.

## Figures and Tables

**Table 1:** The shell thickness and texture properties of HMSNs and solid mesoporous silica nanoparticles (SMSN).

Sample	Shell Thickness ^a^ (nm)	Surface Area ^b^ (m^2^ g^−1^)	Mesopore Volume ^c^ (cm^3^ g^−1^)	Pore Size ^c^ (nm)
HMSN-0.55	52.0 ± 3.4	255	1.12	11.0
HMSN-1.1	67.5 ± 4.1	257	1.07	10.9
HMSN-2.2	92.0 ± 7.7	200	0.72	7.5
SMSN	-	212	0.55	8.4

^a^ Statistic from TEM images; ^b^ BET method; ^c^ BJH model based on the desorption branches.

## Data Availability

The data presented in this study are available on request from the corresponding author.

## Supplementary Materials

The following supporting information can be downloaded at: https://www.mdpi.com/article/10.3390/nano12111940/s1. Synthesis of BaSO_4_ NP-S and BaSO_4_ NP-L; specific enzyme activity measurement; Figure S1. (a) UV-vis spectra of standard catalase solution and (b) the corresponding standard curve; Figure S2. The standard curve for the H_2_O_2_ detection using HRP-TMB method; Figure S3. SEM images of different BaSO_4_ NPs: (a) BaSO_4_ NP-S, (b) BaSO_4_ NP, and (c) BaSO_4_ NP-L, and their corresponding particle size distributions (inset) obtained by the SEM images statistics; Figure S4. EDX spectrum of HMSN; Figure S5. TEM images of BaSO_4_@APF-SiO_2_ with different reaction time TEM images of BaSO_4_@APF-SiO_2_ with different reaction time: 15 min (a1, b1, and c1), 30 min (a2, b2, and c2), 60 min (a3, b3, and c3), and 120 min (a4, b4, and c4). (a), (b), and (c) correspond to different TEOS addition of 0.545, 1.095, and 2.190 mL; Figure S6. (a) TEM image of SMSN; (b) N_2_ sorption isotherm and pore size distribution (inset) of SMSN calculated from desorption branch with BJH model; Figure S7. (a) Standard curve for H_2_O_2_ detection by UV-vis at 240 nm; (b) Enzyme activity of the catalase loaded in HMSN-0.55, HMSN-1.1, HMSN-2.2, and SMSN, and free catalase; (c) the dynamic experiments for the calculation of enzyme activities in (b).
