# Supplementary material for: Dendritic Mesoporous Silica Hollow Spheres for Nano-Bioreactor Application
Source: Nanomaterials (Basel). 2022 Jun 6;12(11):1940. doi: 10.3390/nano12111940 (PMC9182369; doi:10.3390/nano12111940)
Supplement: Supplementary file 1 [file nanomaterials-12-01940-s001.zip › nanomaterials-1740775-supplementary.pdf]

nanomaterials

Supporting Information

Dendritic Mesoporous Silica Hollow Spheres for Nano-Bioreactor Application

Qian Zhang [1], Minying Wu [1], Yuanyuan Fang [1], Chao Deng [2], Hsin-Hui Shen [3], Yi Tang [1,]* and Yajun Wang [2,]*

[1] Department of Chemistry, Fudan University, Shanghai 200433, China; 13110220006@fudan.edu.cn (Q.Z.); 18110220035@fudan.edu.cn (M.W.); 19110220078@fudan.edu.cn (Y.F.)

[2] College of Chemistry & Materials Engineering, Wenzhou University, Wenzhou 325027, China; dengchao@wzu.edu.cn

[3] Department of Materials Science and Engineering, Monash University, Clayton, VIC 3800, Australia; hsin-hui.shen@monash.edu

* Correspondence: yitang@fudan.edu.cn (Y.T.); yajunwang@wzu.edu.cn (Y.W.)

Synthesis of $BaSO_4$ NP-S and $BaSO_4$ NP-L

Based on the synthesis of $BaSO_4$ NP, when using a Ba-EDTA solution of pH 6.0, the sample denoted as **$BaSO_4$ NP-S** was obtained. And when using a Ba-EDTA solution of pH 9.0, 50 mL of Na_2SO_4 and 200 mL of Ba-EDTA, the **$BaSO_4$ NP-L** was obtained. A shown in Figure S1a, most of the $BaSO_4$ NP-S have the shape of an ellipse other than spherical morphology of $BaSO_4$ NP use above, while a few particles of the $BaSO_4$ NP-L are elliptic in Figure S1c. The conditional stability constant of the complex of Ba^{2+} and EDTA ions at pH 9.0 is higher than at pH 6.0, resulting in slower rate of the precipitation of $BaSO_4$ and smaller size. When amplifying the reaction system at pH 6.0, same with $BaSO_4$ NP, once the large volumes of reaction solution was added, a faster rate and local high concentration led to a larger particle and wider size distribution.

Specific Enzyme Activity Measurement

First, immobilized or free catalase was dispersed into a 2 mL of phosphate buffered solution (PBS, 50 mmol L^{-1}, pH 7.0, 25 ℃) containing H_2O_2 (20 mmol L^{-1}), and the concentration of catalase was 2.0 μg mL^{-1}. The absorbance at 240 nm was monitored to track changes in H_2O_2 concentration. The standard curve is shown in Figure S7a.

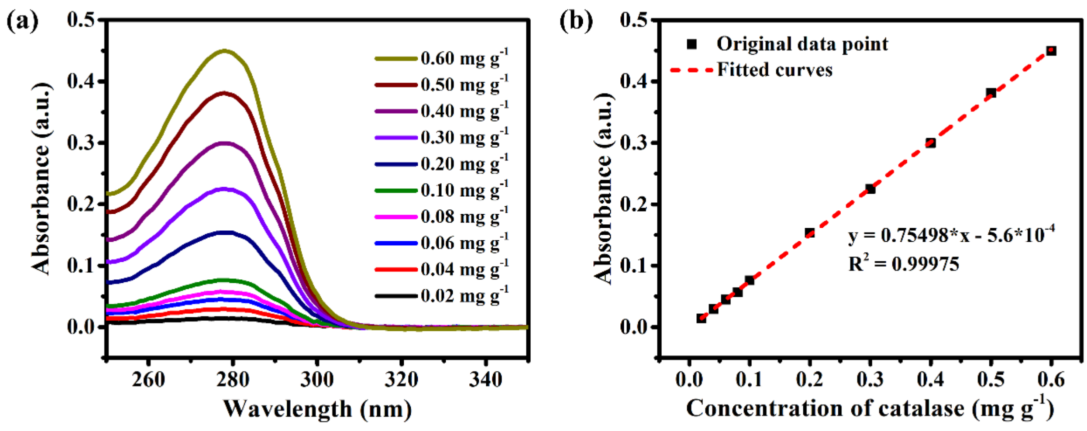

Figure S1. (**a**) UV-vis spectra of standard catalase solution and (**b**) the corresponding standard curve.

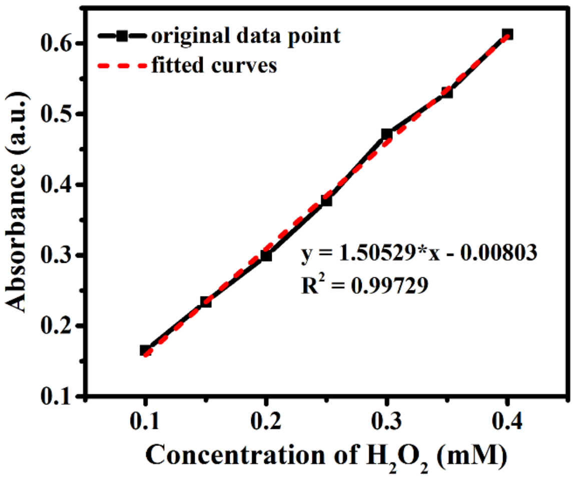

Figure S2. The standard curve for the H_2O_2 detection using HRP-TMB method.

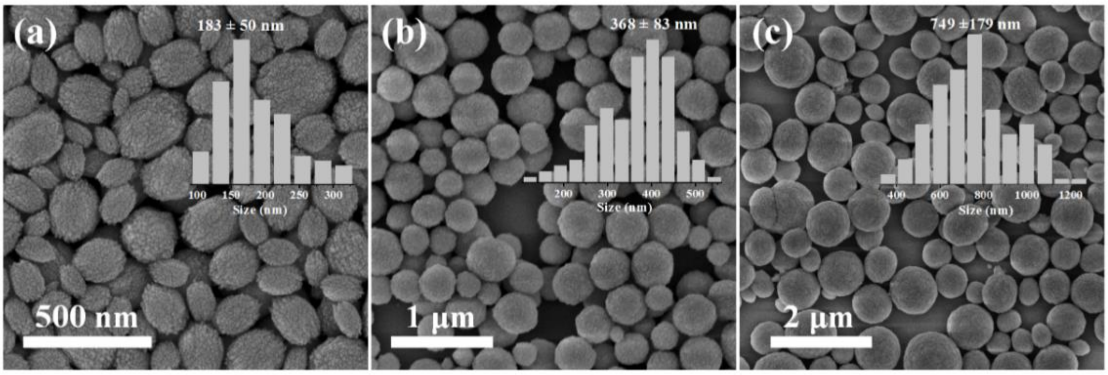

Figure S3. SEM images of different $BaSO_4$ NPs: (**a**) $BaSO_4$ NP-S, (**b**) $BaSO_4$ NP, and (**c**) $BaSO_4$ NP-L, and their corresponding particle size distributions (**insets**) obtained by the SEM images statistics. The numbers of particles counted for $BaSO_4$ NP-S, $BaSO_4$ NP, and $BaSO_4$ NP-L were 109, 127, and 139, respectively.

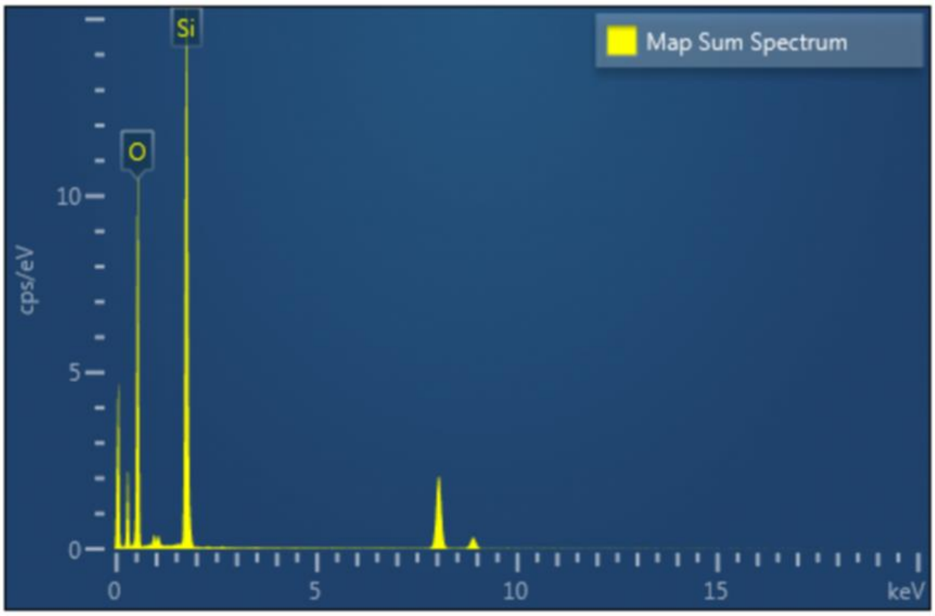

Figure S4. EDX spectrum of HMSN.

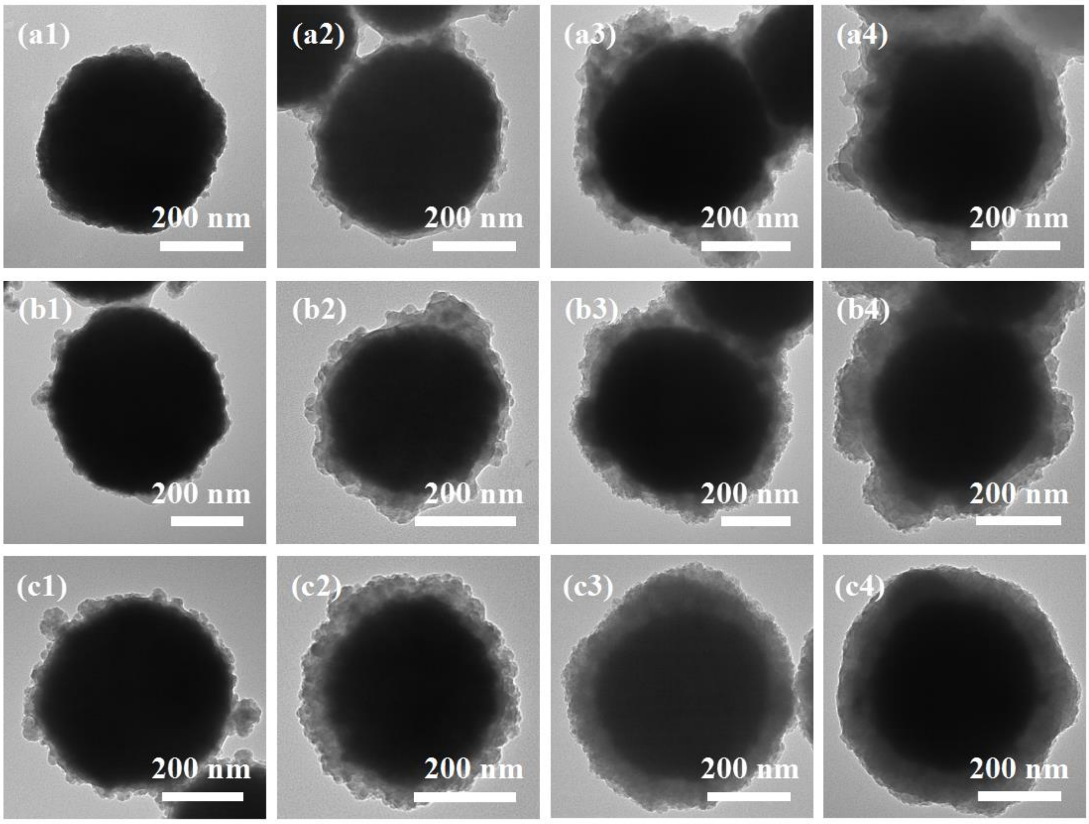

Figure S5. TEM images of $BaSO_4$@APF-SiO_2 with different reaction time: 15 min (**a1, b1,** and **c1**), 30 min (**a2, b2,** and **c2**), 60 min (**a3, b3,** and **c3**), and 120 min (**a4, b4,** and **c4**). (**a**), (**b**), and (**c**) correspond to different TEOS addition of 0.545, 1.095, and 2.190 mL.

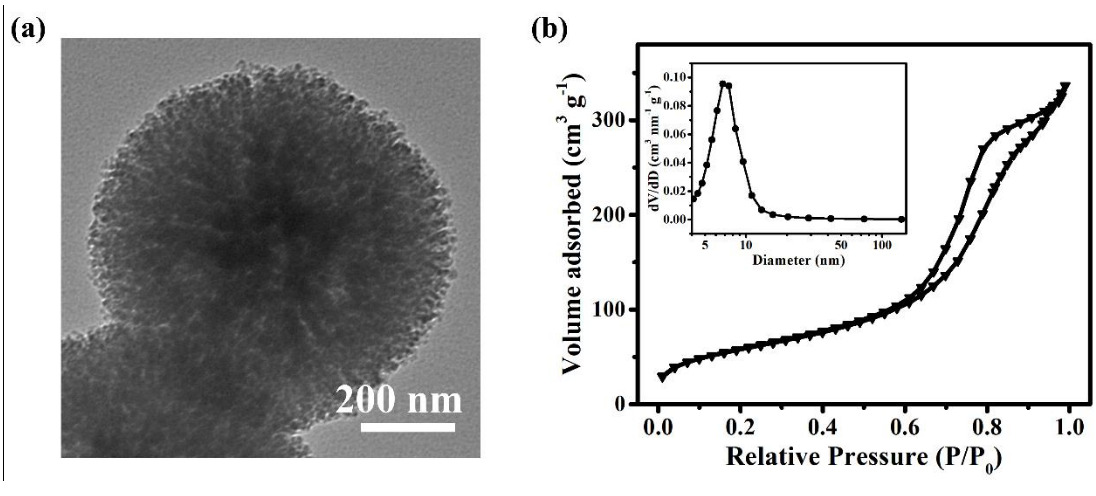

Figure S6. (**a**) TEM image of SMSN; (**b**) N_2 sorption isotherm and pore size distribution (**inset**) of SMSN calculated from desorption branch with BJH model.

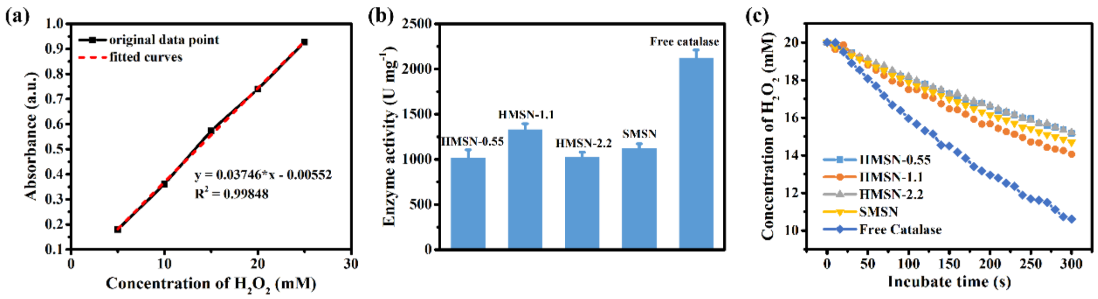

Figure S7. (**a**) Standard curve for H_2O_2 detection by UV-vis at 240 nm; (**b**) Enzyme activity of the catalase loaded in HMSN-0.55, HMSN-1.1, HMSN-2.2, and SMSN, and free catalase; (**c**) the dynamic experiments for the calculation of enzyme activities in (**b**).
